# Increased vegetation disturbance intensity reduces soil nutrients while enhancing microbial network interactions

**DOI:** 10.3389/fmicb.2025.1634424

**Published:** 2025-07-23

**Authors:** Boya Gao, Dan Xiao, Kaixun Yang, Mingming Sun, Shantong Luo, Wei Zhang, Kelin Wang

**Affiliations:** ^1^College of Environment and Ecology, Hunan Agricultural University, Changsha, China; ^2^Institute of Subtropical Agriculture, Chinese Academy of Sciences, Changsha, China; ^3^Huanjiang Agriculture Ecosystem Observation and Research Station of Guangxi, Guangxi Key Laboratory of Karst Ecological Processes and Services, Huanjiang Observation and Research Station for Karst Ecosystems, Chinese Academy of Sciences, Huanjiang, China; ^4^State Key Laboratory for Conservation and Utilization of Subtropical Agro-Bioresources, Guangxi Key Laboratory of Forest Ecology and Conservation, College of Forestry, Guangxi University, Nanning, China; ^5^University of Chinese Academy of Sciences, Beijing, China; ^6^College of Life and Environmental Sciences, Central South University of Forestry and Technology, Changsha, China

**Keywords:** vegetation disturbance, microbial abundance, microbial diversity, microbial interactions, karst ecosystem

## Abstract

**Introduction and methods:**

Vegetation disturbance intensity serves as a critical determinant of changes in soil nutrients and microbial communities. Karst ecosystems are highly fragile, and vegetation degradation has contributed to severe desertification in these regions. However, the specific effects of vegetation disturbance intensity on soil nutrient availability, microbial diversity, and community composition remain poorly understood in karst areas. To address this knowledge gap, this study investigates how varying levels of vegetation disturbance influence soil properties, as well as the diversity, composition, and interactions of bacterial, fungal, and protist communities in a karst ecosystem. The study included four vegetation disturbance intensities: natural vegetation restoration (control) and slight, moderate, and extreme disturbance.

**Results:**

The findings reveal that higher disturbance intensity significantly alters soil nutrient levels, which in turn affects microbial diversity, abundance, community composition, and interspecies interactions. Specifically, increasing vegetation disturbance intensity led to significant declines in soil available nutrients, including nitrate nitrogen (NO₃^−^), available phosphorus (AP), and available potassium (AK). Both slight and moderate disturbances reduced bacterial richness and Shannon diversity, whereas extreme disturbance decreased fungal Shannon diversity compared to the control. Bacterial abundance under moderate and extreme disturbances was significantly lower than that in the control, whereas fungal abundance was significantly higher under extreme disturbance. Although vegetation disturbance reduced soil available nutrients, co-occurrence network analysis revealed greater network complexity under moderate and extreme disturbances, with bacterial-bacterial interactions predominating, alongside enhanced bacterial-fungal and bacterial-protistan interactions. Actinobacteria, Ascomycota, and Chlorophyta emerged as keystone taxa. Pearson correlation analysis identified NO_3_^−^, pH, and soil moisture as primary drivers of microbial abundance and diversity, indicating that higher disturbance intensities reduce bacterial abundance and fungal diversity by limiting soil nutrient availability and moisture. Additionally, community compositions of bacteria, fungi, and protists were significantly correlated with AP and AK.

**Discussion:**

These findings suggest that short-term vegetation recovery following prolonged moderate and extreme disturbances promotes microbial adaptation to nutrient- and moisture-limited conditions through increased microbial interactions, compensating for losses in abundance and diversity. This study provides valuable insights for ecosystem management and soil restoration in degraded karst landscapes.

## 1 Introduction

Karst ecosystems, characterized by soluble carbonate rocks, are distributed globally, including regions such as the Causses in France, the Yucatán Peninsula, the Midwest of the United States. One of the largest and most continuous karst regions spans approximately 0.54 million km^2^ in southwestern China (Wang et al., 2019a). These landscapes are especially vulnerable to anthropogenic disturbances due to their distinctive features, including poor water retention capacity, shallow and discontinuous soil layers, and extensive bedrock exposure (Wang et al., 2019b; Zhang et al., 2020). Therefore, due to the inherent fragility of karst environments, human disturbances often result in more severe ecological consequences, including vegetation degradation, soil erosion, and rocky desertification (Zhang S. et al., 2022). In response to these challenges, restoration efforts such as natural vegetation restoration and afforestation have been implemented to combat desertification (Lu et al., 2022; Qiao et al., 2021). These measures promote nutrient accumulation and improve soil quality by modifying the composition and function of soil microbial communities (Yu et al., 2024). Notably, vegetation loss initiates ecosystem degradation, whereas restoration efforts aim to rehabilitate damaged ecosystems. Disturbances such as deforestation and land reclamation in karst regions reduce aboveground vegetation and underground root systems, leading to decreased biodiversity, lower microbial abundance, and altered microbial interactions (Kang Y. et al., 2024; Qiu et al., 2021). While initiatives like mountain closure for afforestation and the conversion of farmland to forests have shown positive impacts on soil microbial communities, the effects of different disturbance intensities on microbial abundance, diversity, and interactions remain insufficiently understood. A clearer understanding of these responses is critical for developing effective strategies to conserve and restore degraded karst ecosystems.

Soil microorganisms, including bacteria, fungi, and protists, are critical functional groups that influence nutrient cycling and soil fertility in ecosystems (Kiprotich et al., 2025). Bacteria and fungi drive soil carbon (C) dynamics by decomposing organic matter and facilitating C turnover (Geisen et al., 2021), while protists regulate microbial communities by preying on bacteria and fungi (Guo et al., 2018; Oliverio et al., 2020). These trophic interactions position protists as essential regulators within soil microbial networks (Chandarana and Amaresan, 2022). Collectively, the interactions among bacteria, fungi, and protists underpin vital ecosystem functions, including plant productivity and nutrient utilization efficiency (Li et al., 2024; Wang et al., 2024).

Previous study in karst ecosystems have revealed that microbial interactions weaken from the topsoil to deeper soil layers due to reductions in root biomass and soil nutrient availability, highlighting the crucial role of plant root systems and nutrient supply in sustaining microbial interactions (Xiao et al., 2024). Additionally, increased microbial network complexity has been linked to improved ecosystem multifunctionality in karst shrublands (Sun et al., 2025; Xiao et al., 2021). However, the responses of these microbial networks to varying vegetation disturbance intensities are still largely unexplored. This knowledge gap limits the current understanding of how microbial dynamics support ecosystem resilience and multifunctionality under different disturbance regimes in karst landscapes.

Vegetation disturbance intensity significantly influences microbial communities and their interactions (Usman et al., 2024). The frequency, scale, and distribution of disturbances shape microbial responses (Revillini et al., 2022). Previous studies have shown that light grazing intensity can enhance the C sequestration capacity of grassland soils, thereby increasing microbial *α*-diversity (Xun et al., 2018). In contrast, heavy grazing leads to vegetation degradation, lower biomass, and shifts in the soil food web from fungal to bacterial dominance, resulting in reduced fungal abundance and diversity (Cao et al., 2024; Xun et al., 2018). Similarly, deforestation, by removing both above-ground canopies and root systems, reduces organic inputs and suppresses microbial activity and diversity (Faria et al., 2023). Lightly logged forests tend to maintain higher bacterial and fungal diversity, whereas intense logging significantly disrupts fungal communities and alters microbial community composition (Robinson et al., 2024; Tomao et al., 2020). Most notably, increasing vegetation disturbance may intensify microbial competition and reduce microbial network complexity due to declining nutrient availability (Kang H. et al., 2024). Despite these observations, the specific effects of varying vegetation disturbance intensities on the composition, diversity, abundance, and interactions of bacteria, fungi, and protists in karst ecosystems remain poorly understood. Therefore, it is essential to examine how soil microbial communities respond to increasing disturbance and to assess their continued ability to support ecosystem services under these stressors.

This study aims to evaluate the effects of different vegetation disturbance intensities (light, moderate, and extreme) on the abundance, diversity, and interactions of soil microbial communities (bacteria, fungi, and protists) in a karst shrub-grassland ecosystem. Compared with natural vegetation restoration, the microbial responses under different disturbance intensities are analyzed to gain deeper insights into ecosystem recovery and resilience. The hypothesis is that increased vegetation disturbance intensity will lead to a decline in microbial abundance and weaken the interactions among bacteria, fungi, and protists.

## 2 Materials and methods

### 2.1 Study area description

The experimental site is located in Huanjiang County, Guangxi Zhuang Autonomous Region, Southwest China (107° 51′–108° 43′E, 24° 44′–25° 33′N). This region is characterized by a typical karst landscape, featuring peak-cluster depressions and steep slopes. It experiences a subtropical monsoon climate, with mean annual temperatures of 18–20°C and annual rainfall of 1,200–1,600 mm, over 70% of which occurs from May to September. The soil on slopes is thin (10–30 cm) with approximately 30% bedrock exposure, while depressions have thicker soil (50–80 cm). The soil, classified as calcareous, is derived from dolomite bedrock.

### 2.2 Experimental design and soil sampling

Soil samples were collected in July 2023 using a 38-mm diameter auger. Ten soil cores per plot were taken in an S-shaped pattern after removing surface litter. Cores were mixed to form a composite sample, from which stones and visible roots were removed. The samples were passed through a 2 mm sieve and divided into three subsamples. A subsample of each soil was stored at −80°C for DNA extraction, while another portion was kept at 4°C for the determination of ammonium nitrogen (NH₄^+^) and nitrate nitrogen (NO₃^−^) concentrations. For the analysis of soil physicochemical properties, additional subsamples were air-dried for 15 days.

**Figure 1:**
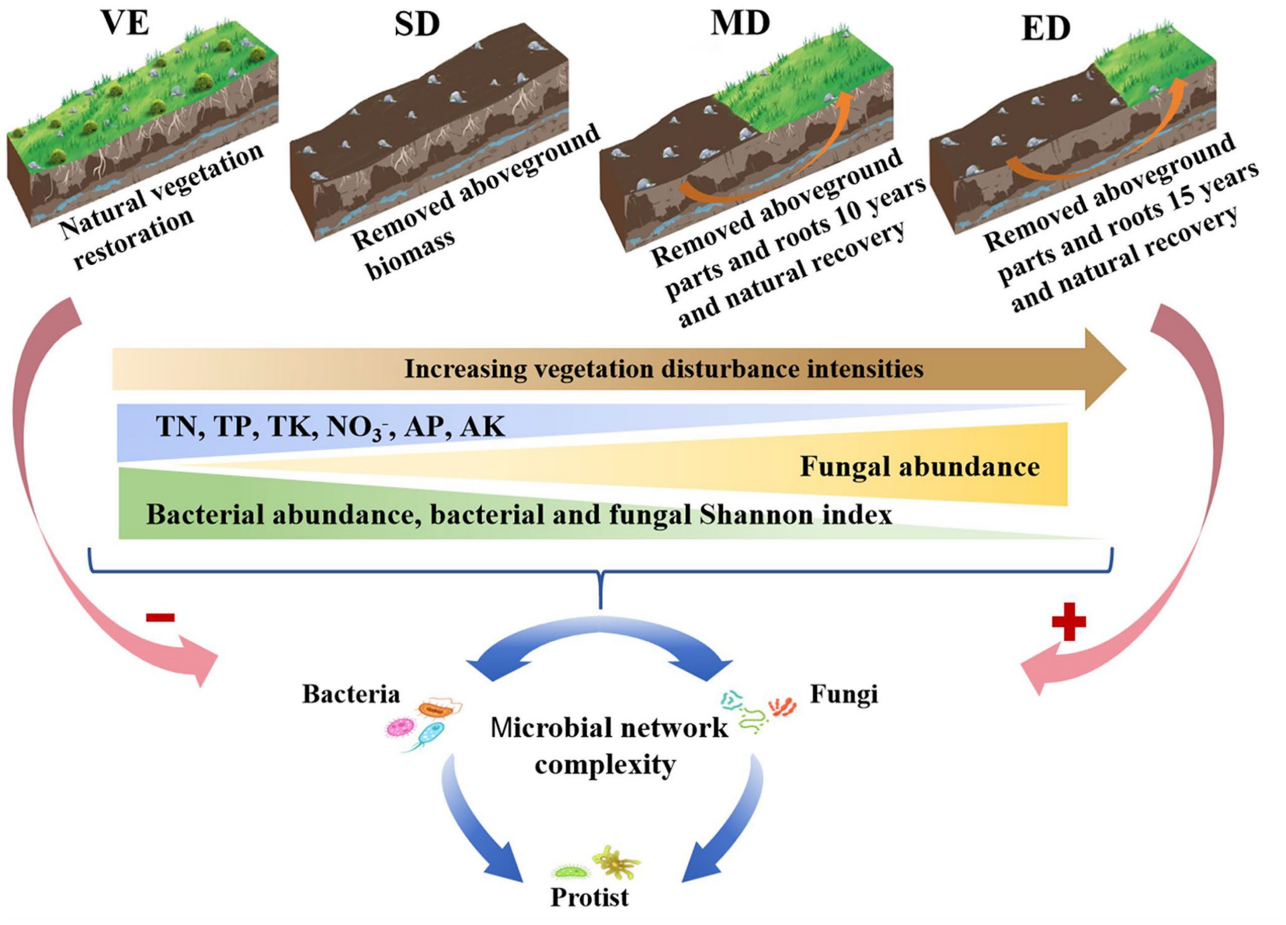
Conceptual model revealing the effect of vegetation disturbance intensities on soil properties, microbial diversities, and microbial network complexity. VE, SD, MD, and ED represent natural vegetation restoration, slight disturbance, moderate disturbance, and extreme disturbance, respectively.

The soil collection sample site experienced severe rocky desertification in its early years due to high population pressure and intensive agricultural activities. Following population relocation in 1985, farming was halted, allowing for natural vegetation recovery dominated by shrub species. By 2006, four experimental treatments representing different vegetation disturbance intensities were established under similar environmental conditions. These treatments included: natural vegetation restoration plot (VE), slightly disturbed plot (SD), moderately disturbed plot (MD), and extremely disturbed plot (ED). Each treatment included six replicate plots (10 m × 10 m). The treatments were characterized as follows (Figure 1):

VE, degraded farmland naturally restored since the end of 2006 and is now dominated by shrub species including *Vitex negundo*, *Pyracantha fortuneana*, and *Zanthoxylum armatum*.
SD, slightly disturbed vegetation; aboveground biomass is removed annually in December, while root systems remain intact since the end of 2006.
MD, moderately disturbed vegetation; both aboveground parts and roots are removed annually since the end of 2006. Natural recovery began in 2016 after 10 years of disturbance.
ED, extremely disturbed vegetation; both aboveground and belowground biomass are removed annually since the end of 2006. Natural recovery began in 2021 after 15 years of disturbance.

### 2.3 Analysis of soil properties

Soil organic carbon (SOC) was measured using dichromate redox colorimetry. Total nitrogen (TN) was determined with an automated elemental analyzer, total phosphorus (TP) via acid digestion, and total potassium (TK) using flame photometry. Ammonium nitrogen (NH_4_^+^) and nitrate nitrogen (NO_3_^−^) were extracted with 0.5 M KCl and quantified using an automatic flow analyzer (Carter and Gregorich, 2006). Available phosphorus (AP) was measured by the molybdenum blue colorimetric method, and available potassium (AK) by neutral ammonium acetate extraction. Soil pH was determined in a 1:2.5 soil-to-water suspension using a Metro 320 pH meter. Exchangeable calcium (Ca^2+^) and magnesium (Mg^2+^) were extracted via ammonium acetate forced exchange and analyzed by inductively coupled plasma atomic emission spectroscopy (ICP-AES) (Gillman and Sumpter, 1986). Soil moisture content was measured by oven-drying soil samples (105°C for 24 h) (Carter and Gregorich, 2006). Soil texture (proportions of clay, silt, and sand) was measured using a laser diffraction particle size analyzer (Mastersizer 2000; Malvern Instruments Ltd., Malvern, UK) (Ryżak and Bieganowski, 2011).

### 2.4 Soil DNA extraction and sequencing

DNA was extracted from soil samples using the Fast DNA SPIN Kit for Soil (MP Biomedicals, USA). DNA concentration and purity were assessed with a NanoDrop 2000 UV–Vis spectrophotometer (Thermo Fisher Scientific, Wilmington, DE, USA). Bacterial 16S rRNA genes were amplified using primers 338F (5′-ACTCCTACGGGAGGCAGCAG-3′) and 806R (5′-GGACTACHVGGGTWTCTAAT-3′) (Guo et al., 2022). Fungal ITS genes were amplified with primers ITS1F (5′-CTTGGTCATTTAGAGGAAGTAA-3′) and ITS2R (5′-GCTGCGTTCTTCATCGATGC-3′) (Xiao et al., 2022b). Protist 18S rRNA genes were amplified using primers TAReuk454FWD1 (5′-CCAGCA(G/C)C(C/T)GCGGTAATTCC-3′) and TAReukREV3 (5′-ACTTTCGTTCTTGAT(C/T/A/G)A-3′) (Maritz et al., 2017). Unique barcode sequences were added to primers to distinguish samples.

PCR reactions were performed in 20 μL volumes containing 0.8 μL primers (10 μM), 0.5 μL diluted DNA (1:10), 0.4 μL FastPfu polymerase, 4 μL 5 × buffer, 0.2 μL BSA, 2 μL dNTPs (2.5 mM), and sterile water. Bacterial 16S rRNA and fungal ITS gene amplification followed: initial denaturation at 95°C for 3 min, 27 (bacteria) or 35 (fungi) cycles of 95°C for 30 s, 55°C for 30 s, and 72°C for 45 s, with a final extension at 72°C for 10 min. Protist 18S rRNA amplification involved: initial denaturation at 95°C for 5 min, 10 cycles of 94°C for 30 s, 57°C for 45 s, and 72°C for 60 s, followed by 25 cycles with gradient annealing (45 s at 45°C, 47°C, 48°C, and 49°C), 94°C for 30 s, 72°C for 60 s, and a final extension at 72°C for 10 min. PCR products were purified via agarose gel electrophoresis, quantified, and sequenced on the Illumina MiSeq PE300 platform (Illumina, San Diego, CA, USA).

Quantitative PCR (qPCR) was used to quantify bacterial 16S rRNA and fungal ITS gene copy numbers using the GeneAmp PCR System 9,700 (Applied Biosystems, Foster City, CA, USA). The 20 μL qPCR mixture contained 0.4 μL of each primer (5 μM), 10 μL of 2 × ChamQ SYBR Color qPCR Master Mix, 2 μL of DNA template, and sterile water. The qPCR protocol included initial denaturation at 95°C for 5 min, followed by 40 cycles of 95°C for 30 s, 56°C for 30 s, and 72°C for 40 s. Amplification efficiency was evaluated, and a standard curve was generated.

### 2.5 Sequence analysis

Sequencing data quality was assessed using fastQC v0.11.9^1^ and multiQC v1.13 (Ewels et al., 2016). Sequences were processed in QIIME2 v2020.2 (Bolyen et al., 2019) and denoised using the DADA2 plugin (Callahan et al., 2016), with truncation lengths of 420 bp (bacteria), 280 bp (fungi), and 400 bp (protists) to generate amplicon sequence variants (ASVs). ASVs were taxonomically annotated using Silva v132 (bacteria), UNITE v6 (fungi), and PR2 (protists) databases. A classifier was trained, and taxonomic information was assigned using the “feature-classifier” plugin (Bokulich et al., 2018). Contaminant sequences were filtered based on taxonomic annotations. To normalize sequencing depth, sequences were rarefied to the smallest sample size using the “rrarefy” function in the R package vegan v2.5–7.

### 2.6 Statistical analysis

All data were tested for normality and homogeneity of variance prior to analysis. The effects of disturbance intensity on soil physicochemical properties, microbial abundance, and diversity were assessed using analysis of variance (ANOVA), followed by Duncan’s multiple range test (*p* < 0.05), implemented via the *agricolae* package v1.3–7 (De Mendiburu, 2019). Non-metric multidimensional scaling (NMDS) based on ASV data was performed using the *metaMDS* function in the *vegan* package v2.6–4 to visualize differences in bacterial, fungal, and protist community composition across disturbance intensities (Oksanen et al., 2015). Pearson correlation analysis was used to assess relationships between microbial abundance/diversity and soil physicochemical properties. The Mantel test was applied to evaluate correlations between microbial community composition and soil environmental variables, using the *mantel* function in the *ape* package v5.7–1 (Paradis et al., 2004). Co-occurrence network analysis was conducted to explore potential interactions among bacterial, fungal, and protist taxa under different disturbance intensities. Significant pairwise associations among ASVs were inferred using the SparCC algorithm with adjusted *p*-values < 0.001(Friedman and Alm, 2012). The resulting networks were visualized using the *igraph* package v1.2.6, adopting the “sphere” layout (Csardi and Nepusz, 2005). All statistical analyses and data visualizations were conducted using R version 4.5.0 (R Core Team, 2020).

## 3 Results

### 3.1 Changes in soil properties along with different vegetation disturbance intensities

**Figure 2:**
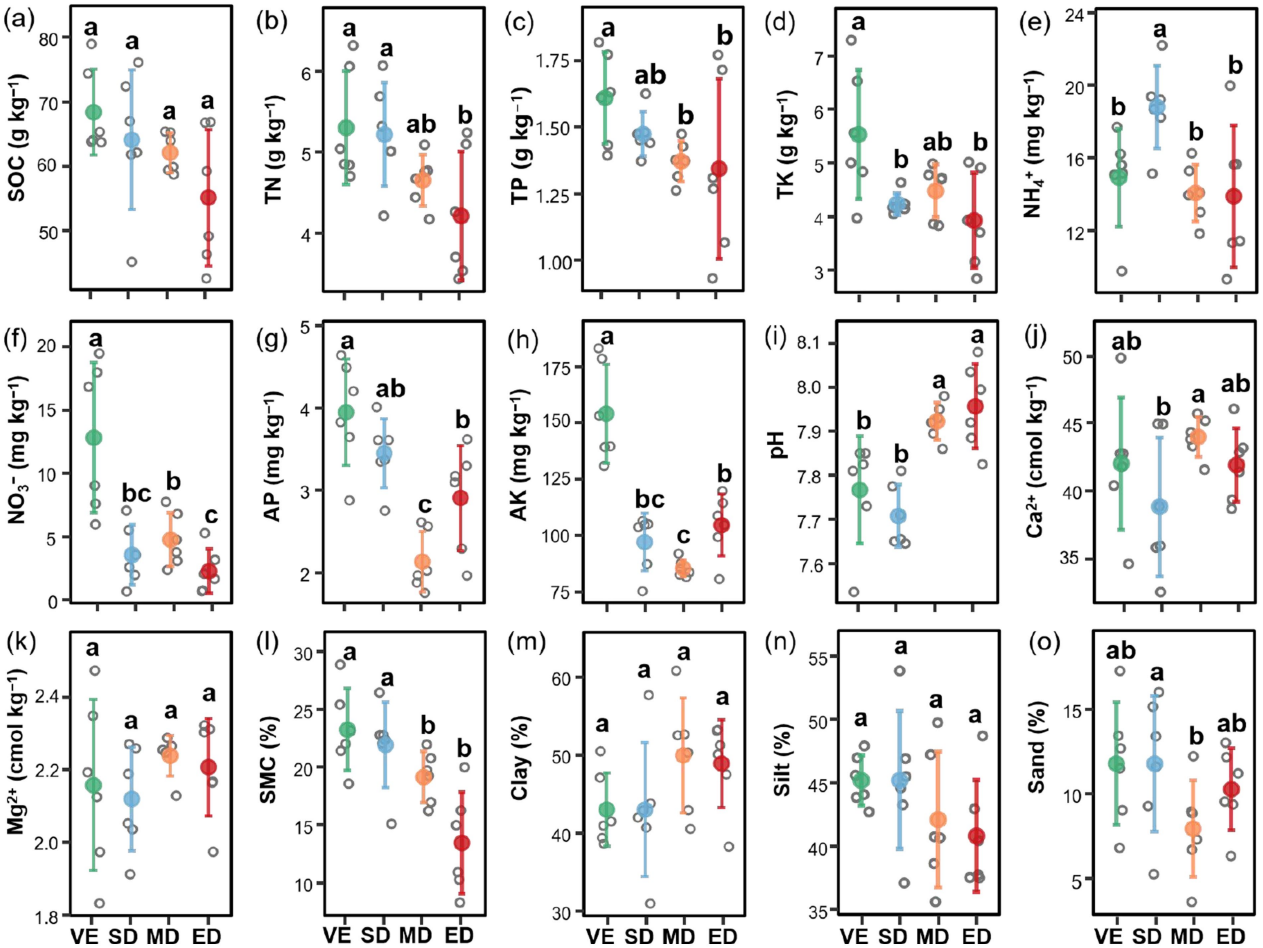
Changes in soil properties under varying vegetation disturbance intensities. Soil properties at different vegetation disturbance intensities. SOC, soil organic carbon **(a)**; TN, total nitrogen **(b)**; TP, total phosphorus **(c)**; TK, total potassium **(d)**; NH_4_^+^, ammonium nitrogen **(e)**; NO_3_^−^, nitrate nitrogen **(f)**; AP, available phosphoru **(g)**; AK, available potassium **(h)**; pH **(i)**; Ca, soil exchangeable Ca^2+^ content **(j)**; Mg, soil exchangeableMg^2+^ content **(k)**; SMC, soil moisture content **(l)**; Clay, soil clay proportion **(m)**; Silt, soil silt proportion **(n)**; Sand, soil sand proportion **(o)**. VE, SD, MD, and ED represent natural vegetation restoration, slight disturbance, moderate disturbance, and extreme disturbance, respectively. Different lowercase letters indicate significant differences among treatments (*p* < 0.05).

Vegetation disturbance significantly affected soil properties. Specifically, compared with natural vegetation restoration, all three disturbance intensities significantly reduced NO_3_^−^ and AK content but had no significant impact on SOC or Mg^2+^ content. In addition, TN (except in MD), TP, TK (except in MD), AP, and soil moisture content were significantly lower under moderate and extreme disturbance than under natural vegetation restoration (*p* < 0.05). The most pronounced decreases occurred under extreme disturbance, with reductions of 45.73% for TN, 48.87% for TP, 61.06% for TK, 57.64% for AP, and 71.30% for soil moisture content relative to natural vegetation restoration. Conversely, soil pH was significantly higher under moderate and extreme disturbance compared to slight disturbance and control (*p* < 0.05) (Figure 2).

### 3.2 Abundance and diversity of bacteria, fungi, and protists

**Figure 3:**
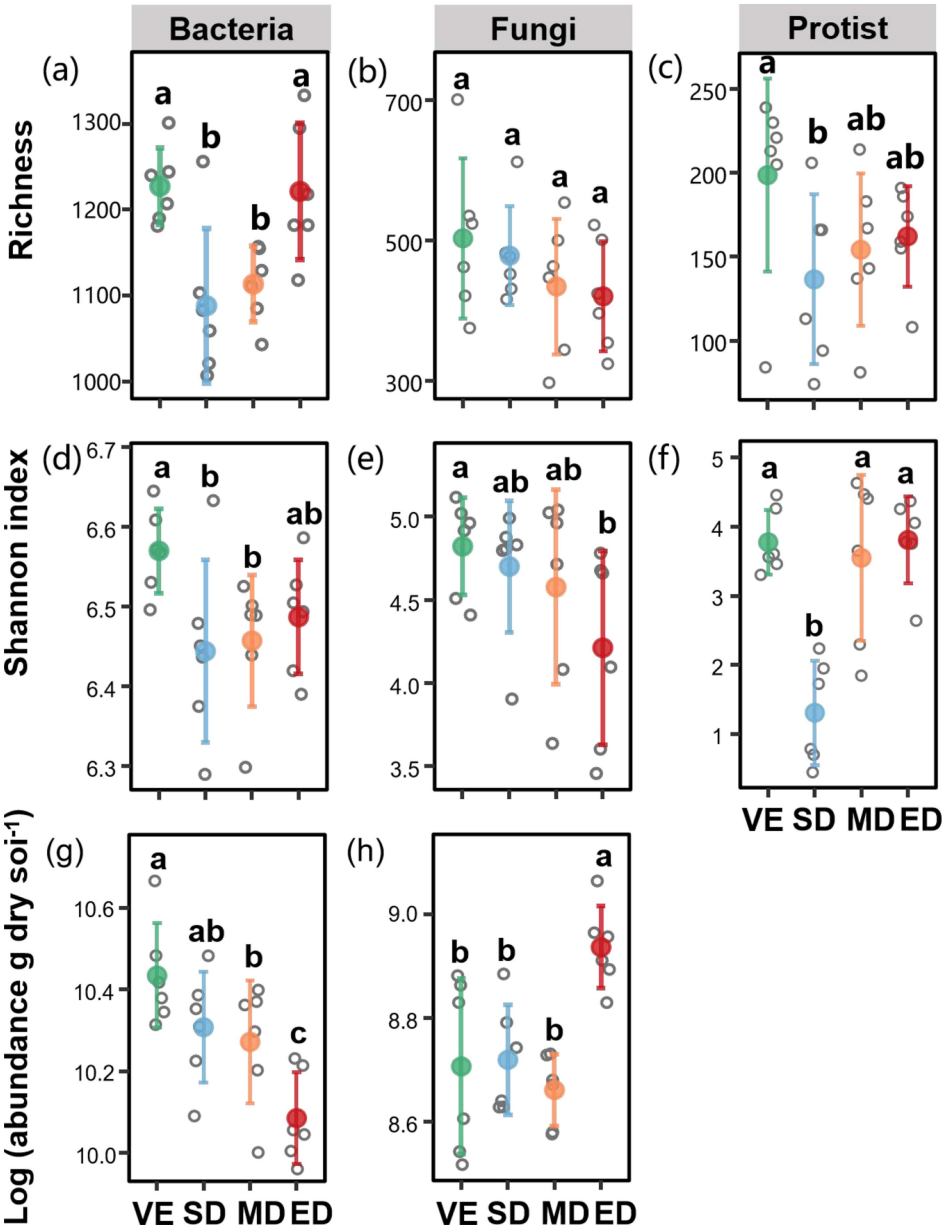
Changes in richness, Shannon index, and abundances of bacteria, fungi, and protists under varying vegetation disturbance intensities. Soil bacterial, fungal, and protistan richness **(a–c)**; Shannon index **(d–f)**; and bacterial and fungal abundances (gene copies) **(g,h)** across varying vegetation disturbance intensities. VE, SD, MD, and ED represent natural vegetation restoration, slight disturbance, moderate disturbance, and extreme disturbance, respectively. Different lowercase letters indicate significant differences among treatments (*p* < 0.05).

Bacterial richness and Shannon diversity were significantly lower under slight and moderate disturbance compared to control (*p* < 0.05), with slight disturbance decreased by 24.95 and 5.22%, and moderate disturbance decreased by 21.68 and 5.36%, respectively. Fungal diversity tended to decline with increasing disturbance, with a significantly lower Shannon index observed under extreme disturbance (*p* < 0.05), which was decreased by 32.46% compared with the control. Under slight disturbance, both protist richness and the Shannon index were significantly reduced (by 60.74 and 89.96%, respectively) compared to the control. Moreover, moderate and extreme disturbances resulted in a significant decrease in bacterial abundance compared to the control. In contrast, fungal abundance was significantly higher under extreme disturbance than under slight, moderate, and control conditions (*p* < 0.05) (Figure 3).

### 3.3 Community compositions of bacteria, fungi, and protists

**Figure 4:**
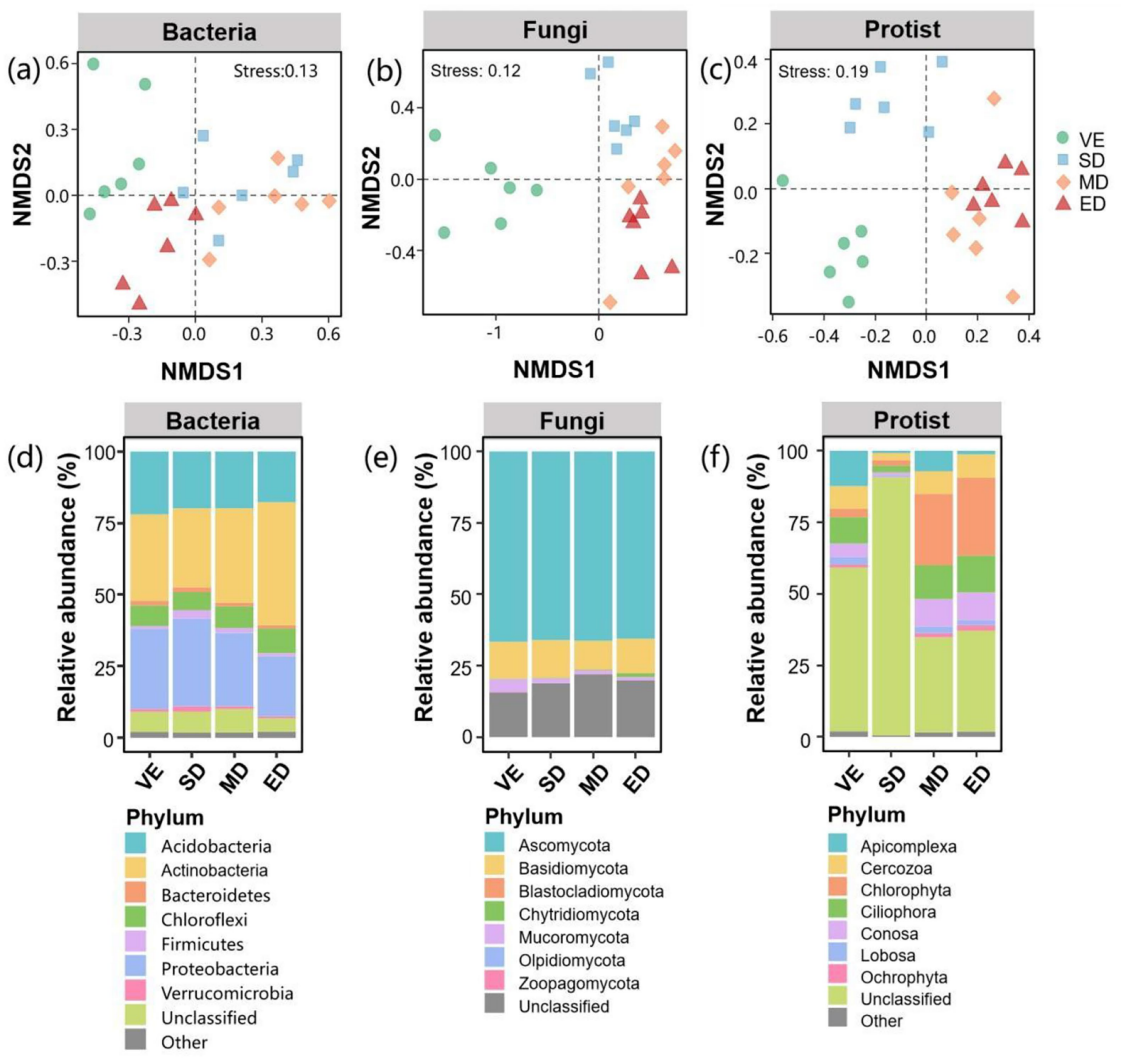
Compositions of bacterial, fungal, and protistan communities under varying vegetation disturbance intensities. Bacterial, fungal, and protistan community compositions based on non-metric multidimensional scaling (NMDS) analysis **(a–c)**; microbial community compositions at the phylum level **(d–f)**. VE, SD, MD, and ED represent natural vegetation restoration, slight disturbance, moderate disturbance, and extreme disturbance, respectively.

Non-metric multidimensional scaling (NMDS) analysis revealed that the community compositions of bacteria, fungi, and protists differed significantly among the disturbance treatments and the control (Figures 4a,b,c). At the phylum level, bacterial communities were dominated by Actinobacteria (27.6–42.9%), Proteobacteria (20.9–30.6%), and Acidobacteria (17.7–21.9%) (Figure 4d). The relative abundance of Actinobacteria was significantly higher under extreme disturbance than in the other treatments. In contrast, Proteobacteria abundance was significantly lower under extreme disturbance than under slight disturbance and control (Supplementary Figure S1).

For fungi, Ascomycota (65.5–66.6%) and Basidiomycota (10.1–13.2%) were the dominant taxa (Figure 4e). Disturbance treatments had no significant effect on their relative abundances (Supplementary Figure S2). Protist communities were dominated by Chlorophyta (1.9–27.2%), Ciliophora (2.4–12.9%), and Conosa (1–9.7%) (Figure 4f). The relative abundance of Chlorophyta was significantly higher under moderate and extreme disturbance than under light disturbance and control. In contrast, the relative abundances of Ciliophora, Conosa, and Lobosa were significantly lower under slight disturbance than in other treatments (Supplementary Figure S3).

### 3.4 Microbial co-occurrence networks involving bacteria, fungi, and protists

**Figure 5:**
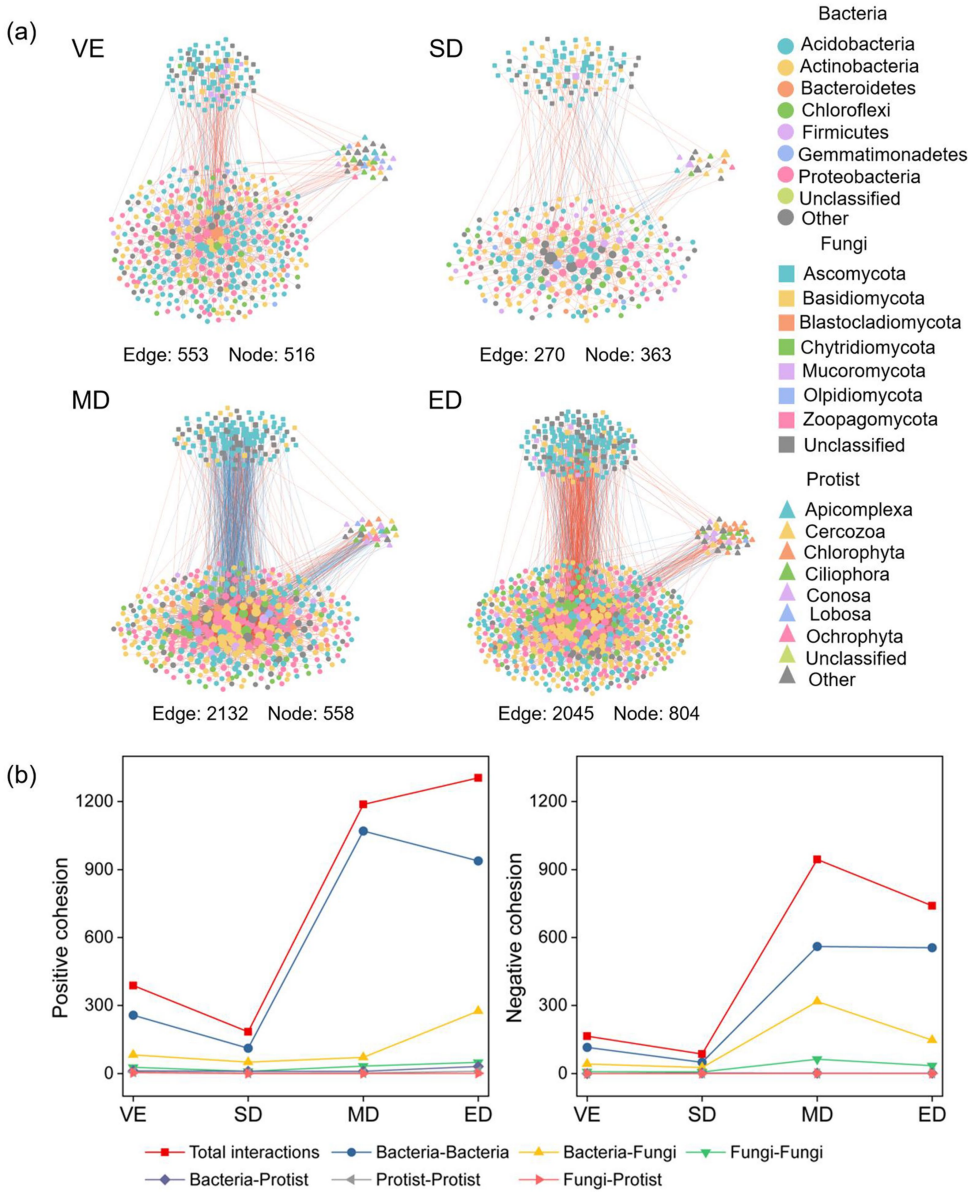
Co-occurrence network patterns of bacteria, fungi, and protists under varying vegetation disturbance intensities **(a)**. The positive and negative cohesion among nodes in microbial co-occurrence networks under varying vegetation disturbance intensities **(b)**. Edges between node pairs represent positive (orange) or negative (blue) interactions. Node size indicates the number (or proportion) of connections shared with other taxa. VE, SD, MD, and ED represent natural vegetation restoration, slight disturbance, moderate disturbance, and extreme disturbance, respectively.

Networks under moderate and extreme disturbance showed greater complexity, with increased numbers of nodes, edges, and higher connectivity than those under natural vegetation restoration. In all networks, bacterial communities consistently had more nodes than fungal or protist communities (Figure 5; Supplementary Table S1).

Across treatments, the dominant bacterial phyla were Actinobacteria, Acidobacteria, and Proteobacteria, while Ascomycota and Mucoromycota dominated among fungi. Among protists, Chlorophyta, Ciliophora, and Conosa were the major groups. These taxa served as keystone species with high connectivity. Bacteria–bacteria interactions were the most prevalent (59.63–76.50%), followed by bacteria–fungi (18.25–28.15%) and bacteria–protist interactions (4.11–6.33%). Under moderate and extreme disturbance, the number of interactions increased markedly, including Actinobacteria–Actinobacteria, Proteobacteria–Actinobacteria, Actinobacteria–Ascomycota, Proteobacteria–Ascomycota, Actinobacteria–Ciliophora, and Proteobacteria–Conosa, compared with control conditions (Figure 5).

### 3.5 Relationships between microbial traits and soil properties

**Figure 6:**
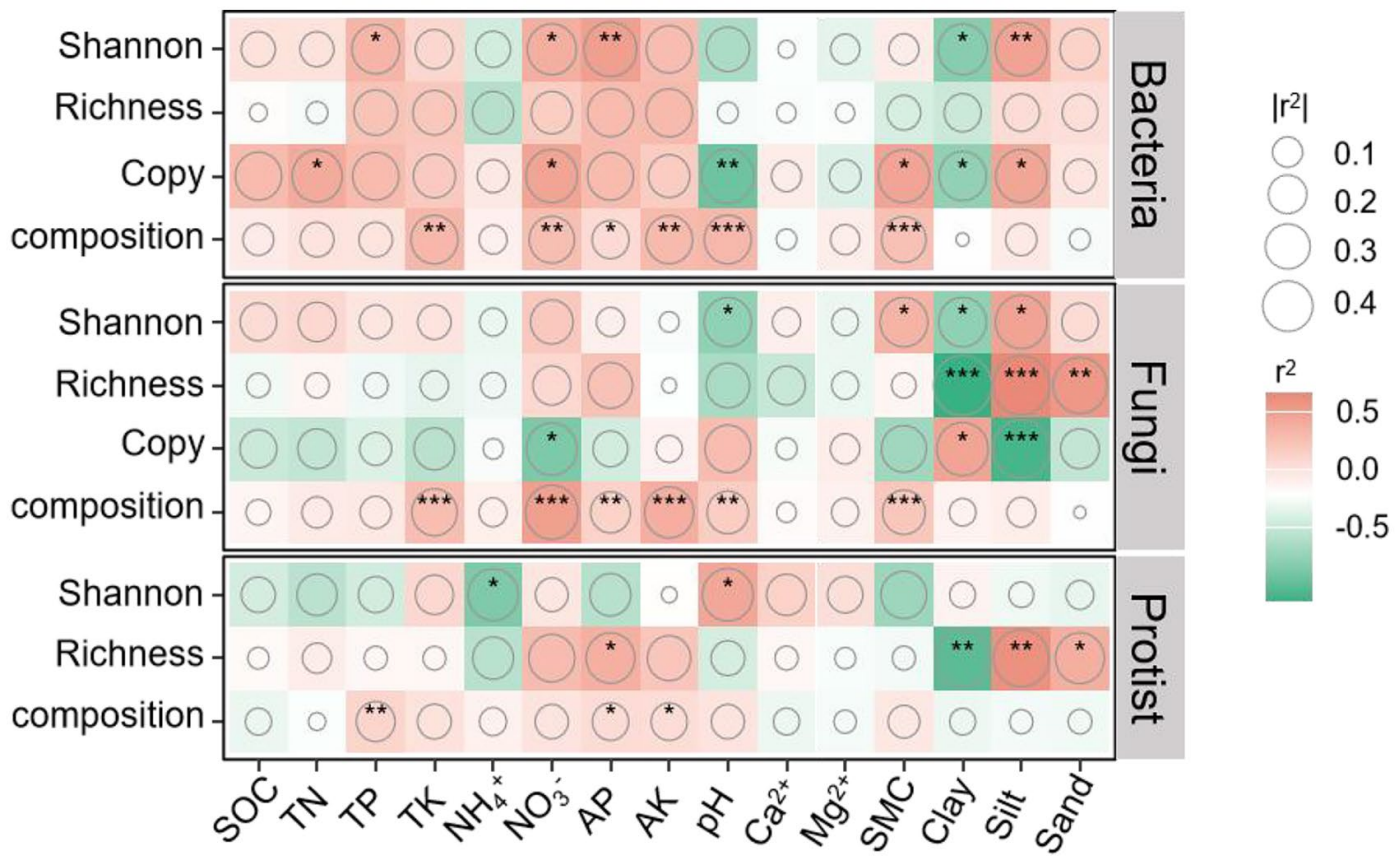
Pearson’s correlations between bacterial, fungal, and protistan community profiles and soil properties. The color intensity and size of the circles represent the strength of the correlation. Asterisks (*, **, ***) indicate significance levels of *p* < 0.05, *p* < 0.01, and *p* < 0.001, respectively.

Pearson correlation analysis showed that bacterial abundance and Shannon index were positively correlated with NO_3_^−^, TN (abundance only), soil moisture content (abundance only), and silt, but negatively correlated with clay and pH (abundance only). Fungal diversity was positively correlated with silt but negatively with clay. Fungal abundance showed a positive correlation with clay and a negative correlation with NO_3_^−^. For protists, Shannon index was positively correlated with pH and negatively with NH_4_^+^, while richness was negatively correlated with clay and positively with silt. Additionally, community compositions of bacteria, fungi, and protists were significantly correlated with AP, AK, and soil moisture content (except for protists), pH (except for protists), and NO_3_^−^ (except for protists) (Figure 6). Therefore, increasing vegetation disturbance intensity leads to reductions in bacterial and fungal diversity, as well as bacterial abundance, primarily due to decreased soil nutrient availability and moisture content. In contrast, higher disturbance intensity appears to stimulate enhanced microbial interactions, potentially as a compensatory response to nutrient and moisture limitations.

## 4 Discussion

### 4.1 Effects of disturbance intensities on the abundance and diversity of bacteria, fungi, and protists

Consistent with the hypothesis, vegetation disturbance reduced bacterial diversity and abundance compared to natural vegetation restoration (control), with the lowest bacterial abundance observed under extreme disturbance. This aligns with previous studies (Li et al., 2025) and may result from reduced litter inputs and root biomass, which decrease organic matter and nutrient availability, limiting microbial substrates (Feng et al., 2024a; Feng et al., 2024b). Soil pH is widely recognized as an important predictor of microbial abundance (Mitsuta et al., 2025). Compared to natural vegetation restoration, the reduction in organic acids (normally released by plant roots) under moderate and extreme disturbances resulted in a significant increase in soil pH (Yang et al., 2020). Although earlier studies found higher bacterial abundance in weakly alkaline karst soils relative to acidic non-karst soils (Cheng et al., 2023; Wang et al., 2021), results showed a negative correlation between soil pH and bacterial abundance. This suggests that in alkaline soils, the effect of pH changes on bacterial abundance may be mediated by other factors, such as nutrient availability (Lopes et al., 2021). Bacteria are also particularly sensitive to changes in soil nutrient and water availability (Chen et al., 2022; Yang et al., 2021). Long-term vegetation disturbance can disrupt the soil environment and reduce its suitability for bacterial growth (Zhang Y. et al., 2022). Increasing disturbance intensities led to declines in soil nutrients (e.g., TN, NO_3_^−^, AP, AK) and moisture content. Bacteria generally thrive in nutrient-rich environments, and under short-term recovery following disturbance, soil conditions are not fully restored, resulting in reduced bacterial abundance and diversity (Chen L. et al., 2021; Nguyen J. et al., 2021). This is supported by the significant positive correlation between bacterial abundance and diversity with NO_3_^−^ and soil moisture content. Thus, Vegetation disturbance markedly decreases bacterial abundance and diversity, closely linked to changes in soil pH, nutrients, and moisture.

Fungi, in contrast, are more efficient at utilizing complex compounds (e.g., cellulose and lignin) and are more tolerant of low-nutrient and arid conditions (Singh et al., 2024; Wang and Kuzyakov, 2024). Studies in karst regions have shown that fungi outperform bacteria in decomposing organic matter under nutrient-limited conditions (Xiao et al., 2022a). In this study, fungal abundance was significantly higher under extreme disturbance compared to the control, slight, and moderate disturbances. This aligns with observations from high-altitude regions such as the Tibetan Plateau, where fungi efficiently degrade recalcitrant C sources (Li et al., 2023). However, the fungal Shannon index was significantly lower under extreme disturbance than in the control. This may be due to substantially reduced soil nutrient contents (TN, TP, TK, AP) and soil moisture in areas with extreme disturbance, favoring the dominance of specific fungal taxa capable of degrading recalcitrant compounds, while reducing overall fungal diversity (Liu et al., 2023; Zhang X. et al., 2025). Consequently, fungal abundance increases under extreme disturbance, but diversity decreases, reflecting their adaptability to nutrient-poor environments.

Protists play critical roles in microbial food webs, often acting as predators of bacteria and fungi (Zhang A. et al., 2025). In this study, bacterial richness and diversity were significantly lower under moderate disturbance compared to the control, leading to reduced prey availability and, consequently, lower protist diversity. Additionally, protist Shannon index was negatively correlated with NH_4_^+^, suggesting that NH_4_^+^ inhibits protist growth, as supported by prior studies (Xu L. et al., 2025; Yang et al., 2019). Slight disturbance, characterized by removal of aboveground vegetation while retaining roots, likely led to NH_4_^+^ accumulation from microbial decomposition of root residues in the absence of plant uptake (Zhu et al., 2021). This effect mirrors observations in agricultural soils with high N enrichment (Wang et al., 2025a). Overall, protist diversity declines due to reduced prey availability and nutrient accumulation, underscoring their critical role in microbial food webs (Guo et al., 2023; Xu H. et al., 2025).

### 4.2 Effects of disturbance intensities on the community composition of bacteria, fungi, and protists

Vegetation disturbance intensity significantly altered microbial community composition. For bacteria, extreme disturbance significantly increased the relative abundance of Actinobacteria while decreasing Proteobacteria compared to the control and light disturbance. This shift likely reflects the oligotrophic nature of Actinobacteria, which can degrade recalcitrant compounds like cellulose and lignin (Bao et al., 2021; Mitra et al., 2022), while Proteobacteria, which prefer nutrient-rich conditions (Chen L. F. et al., 2021), are inhibited by reduced soil water content and easily degradable C sources under extreme disturbance (Huang et al., 2021). For fungi, the dominant phyla Ascomycota and Basidiomycota showed no significant changes in relative abundance across disturbance intensities. This may be because fungi can access nutrients more effectively via mycelial growth, reducing the adverse impacts of disturbance (Napitupulu, 2025; Wahab et al., 2023).

For protists, the relative abundance of Chlorophyta was significantly higher under moderate and extreme disturbances than under slight disturbance and control. This aligns with findings in degraded meadows (Zhao et al., 2023), where autotrophic Chlorophyta can grow through photosynthetic carbon fixation under nutrient-poor conditions (Kang E. J. et al., 2024; Nguyen B. T. et al., 2021). In contrast, the relative abundances of Ciliophora, Conosa, and Lobosa were significantly lower under light disturbance compared to other treatments. Ciliophora, a sensitive consumer, may have declined due to reduced prey availability (Qu et al., 2022), while Conosa and Lobosa, typically decomposers, may have been limited by reduced organic matter content (Sun et al., 2024). Overall, microbial taxa display distinct adaptive responses to different vegetation disturbance intensities, with disturbance serving as a filter that selects for specific microbial communities (Hoang et al., 2024; Santillan et al., 2019). Taken together, disturbance intensity induces specific shifts in microbial community composition, reflecting unique adaptive strategies of different microbial groups (Philippot et al., 2021).

### 4.3 Effects of disturbance intensities on microbial co-occurrence network

Co-occurrence network analysis reveals potential microbial interactions and provides insights into structural changes within microbial communities under varying disturbance levels (Ona et al., 2025; Wu et al., 2023). In this study, microbial networks under moderate and extreme disturbances exhibited higher numbers of nodes, edges, average degree, clustering coefficient (transitivity), and mean betweenness centrality than those under light disturbance and control. This indicates that microbial networks became more complex under higher disturbance intensities, contrary to the original hypothesis. Bacteria had more nodes and edges than fungi and protists in all networks, suggesting they formed the core of microbial interactions, particularly under moderate and extreme disturbances.

First, increasing vegetation disturbance results in nutrient loss and unstable soil physicochemical properties during early restoration stages, prompting microbes to compete for limited resources such as C and N, thereby intensifying microbial interactions (Du et al., 2023; Wu et al., 2021). Both positive and negative microbial interactions increased with disturbance intensity. Despite reductions in bacterial abundance and fungal diversity, the surviving keystone taxa may assume more crucial ecological roles, maintaining ecosystem function through competitive or cooperative interactions (Liu et al., 2022). Second, long-term disturbance alters soil structure, moisture, and temperature, exerting stress on microbes. During the initial recovery phase, limited plant cover and unstable conditions encourage microbes to form adaptive interactions to occupy new ecological niches and enhance community stability (Du et al., 2025; Hu et al., 2022). Third, the colonization of fast-growing herbaceous or shrubby plants during early recovery may actively recruit beneficial microbes through root exudates, further promoting microbial interactions (Williams and de Vries, 2020). Consequently, increasing disturbance intensity enhances the complexity of microbial co-occurrence networks, suggesting that microbial interactions are strengthened under resource limitation and environmental stress to maintain community stability (Santillan et al., 2025; Yuan et al., 2021).

Key taxa, including Actinobacteria, Acidobacteria, Proteobacteria (bacteria), Ascomycota, Mucoromycota (fungi), and Chlorophyta, Ciliophora, Conosa (protists), acted as keystone species. Actinobacteria, Proteobacteria, and Ascomycota, fast-growing and enzymatically versatile, adapt quickly to environmental changes. Acidobacteria, Mucoromycota, and Chlorophyta, oligotrophic and capable of degrading complex substrates, play key roles in C cycling under resource scarcity (Chai et al., 2024; Yang et al., 2023). Notably, interactions between Actinobacteria and Proteobacteria were strongest under moderate disturbance, suggesting enhanced metabolic complementarity and symbiosis under moderate stress. Therefore, disturbance enhances microbial network complexity, with bacteria as central players and keystone taxa sustaining ecosystem functions through adaptive interactions (Rawstern et al., 2024; Wang et al., 2024).

### 4.4 Implications for vegetation recovery

Soil and vegetation shape microbial communities (Wang et al., 2025b). Increased disturbance reduces root exudates, lowering organic matter and nutrient inputs, which decreases bacterial abundance and fungal diversity while strengthening microbial interactions. After long-term disturbance, short-term vegetation restoration creates an unstable soil ecosystem (Liu et al., 2025). Resource scarcity and environmental stress prompt microorganisms to adapt by enhancing interactions, compensating for reduced abundance and diversity (Dal Bello et al., 2021; Wani et al., 2022). This dynamic significantly impacts bacterial communities, leading to abundance declines. These findings suggest that short-term restoration strategies should prioritize fostering microbial interactions over solely enhancing abundance and diversity to promote rapid ecosystem recovery (Bhatia et al., 2023; Singh Rawat et al., 2023). In karst shrub-grass ecosystems, nutrient cycling driven by microbial interactions under varying disturbance intensities warrants particular attention.

## 5 Conclusion

This study systematically demonstrates that vegetation disturbance intensity significantly affects soil microbial communities by altering their diversity, abundance, composition, and interactions. Increased disturbance, compared to natural vegetation restoration, significantly reduced bacterial abundance and fungal diversity by limiting soil nutrient availability (e.g., TN, NO_3_^−^) and moisture. In contrast, moderate and extreme disturbances increased the complexity of microbial co-occurrence networks, driven by enhanced interactions among bacteria, fungi, and protists. These findings highlight adaptive strategies of microbial communities under nutrient- and moisture-limited conditions. Importantly, short-term vegetation recovery following moderate or extreme disturbances may promote microbial adaptation through intensified interactions, compensating for declines in abundance and diversity. This study provides a valuable foundation for understanding microbial adaptation to environmental stress and offers insights for ecosystem management and soil restoration in DISTURBED karst landscapes.

## Data Availability

The data presented in the study are deposited in the Figshare repository, accession number https://doi.org/10.6084/m9.figshare.29517656.v1.

## Footnotes

^1^ http://www.bioinformatics.babraham.ac.uk
