# Supplementary material for: Increased vegetation disturbance intensity reduces soil nutrients while enhancing microbial network interactions
Source: Front Microbiol. 2025 Jul 23;16:1634424. doi: 10.3389/fmicb.2025.1634424 (PMC12325223; doi:10.3389/fmicb.2025.1634424)
Supplement: Supplementary file 1 [file Data_Sheet_1.PDF]

Supplementary Material

1 Supplementary Tables

Table S1 Topological properties of co-occurrence networks among bacterial, fungal, and protistan taxa under different vegetation disturbance intensities (as shown in Fig. 4).

Topological properties	Vegetation disturbance intensities			
	VE	SD	MD	ED
Network transitivity	0.06	0.06	0.25	0.12
Network degree	2.14	1.49	7.64	5.09
Mean betweenness	321	19	383	987
Connectivity	3.77	0.80	25.74	18.03
Number of nodes	516	363	558	804
Number of edges	553	270	2132	2045
Bacterial nodes	387	259	422	589
Fungal nodes	95	88	111	173
Protist nodes	34	16	25	42

2 Supplementary Figures

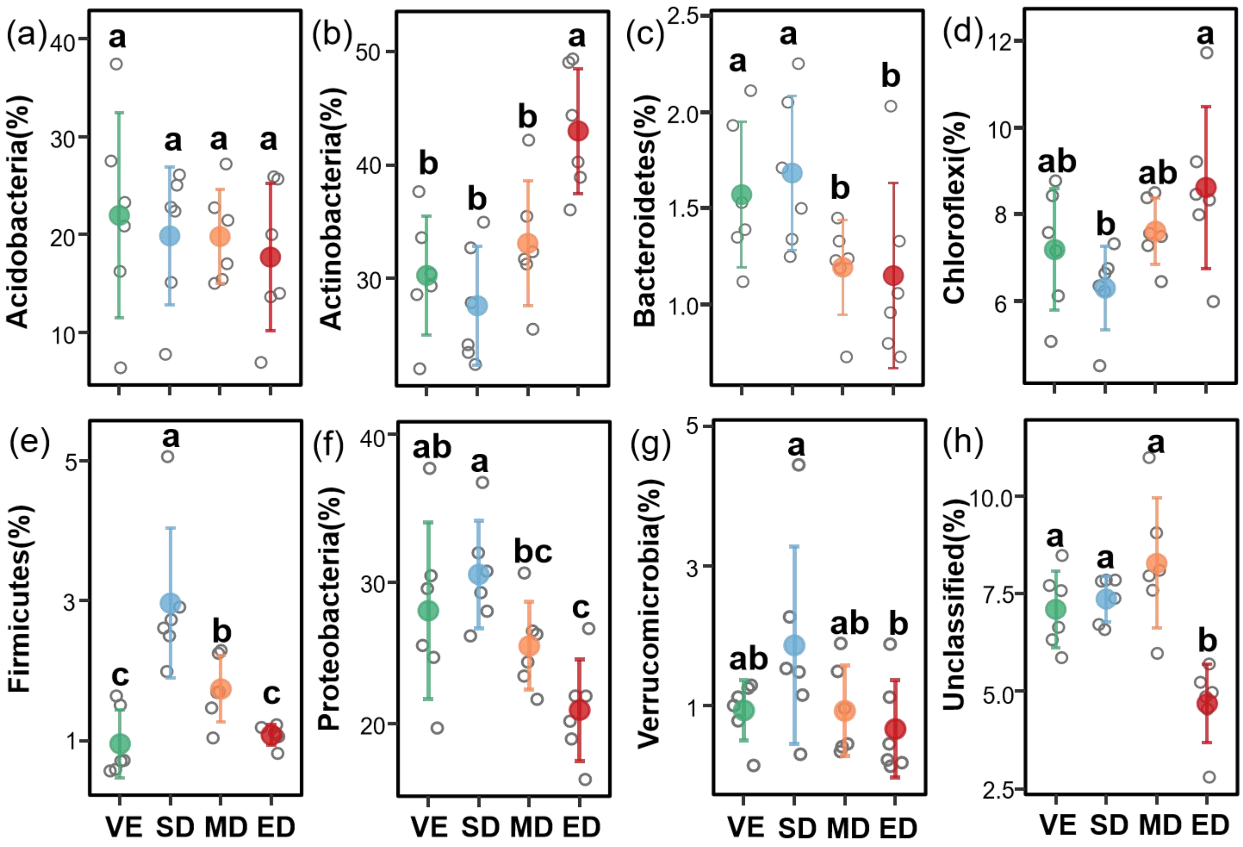

Supplementary Figure 1. Change in the relative abundance of bacterial for each phylum.

VE, SD, MD, and ED represent natural vegetation restoration, slight disturbance, moderate disturbance, and extreme disturbance, respectively. Different lowercase letters indicate significant differences among treatments ($p < 0.05$).

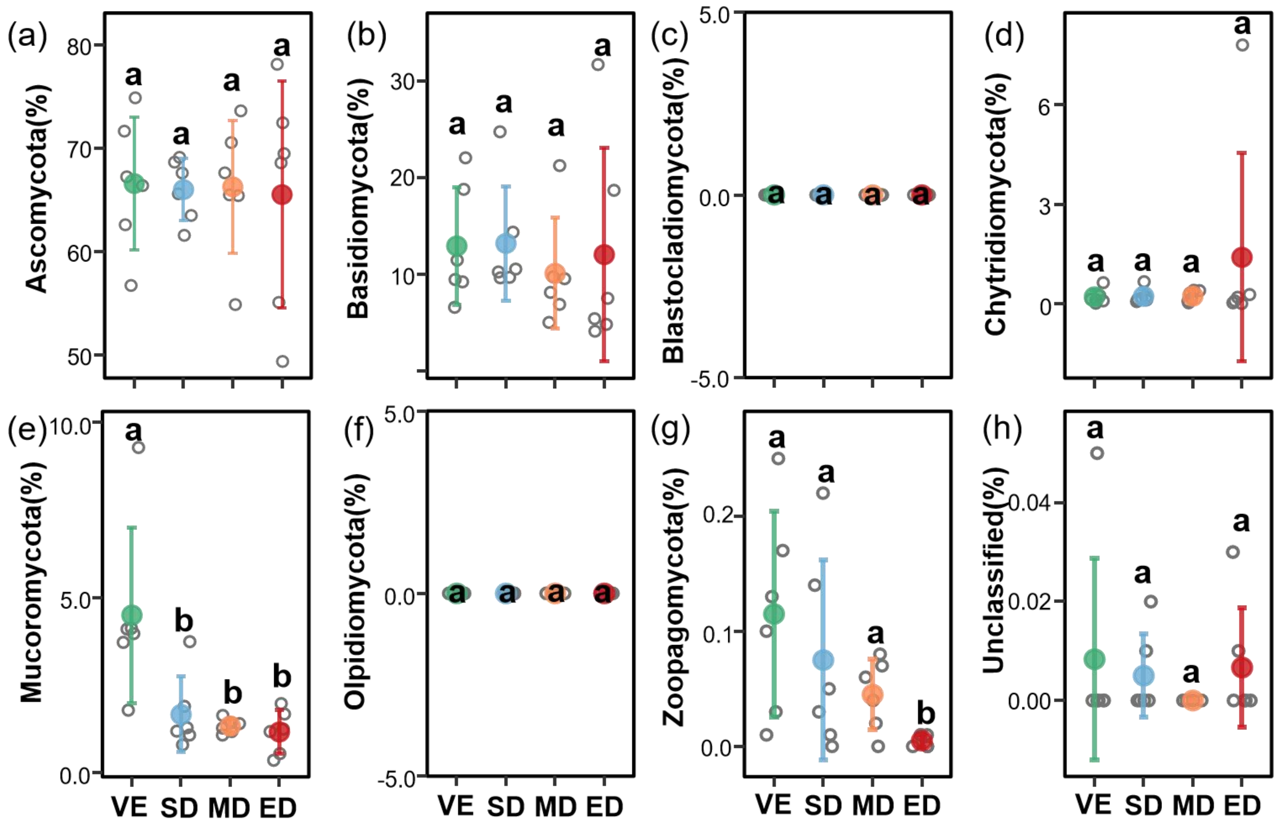

Supplementary Figure 2. Change in the relative abundance of fungal for each phylum.

VE, SD, MD, and ED represent natural vegetation restoration, slight disturbance, moderate disturbance, and extreme disturbance, respectively. Different lowercase letters indicate significant differences among treatments ($p < 0.05$).

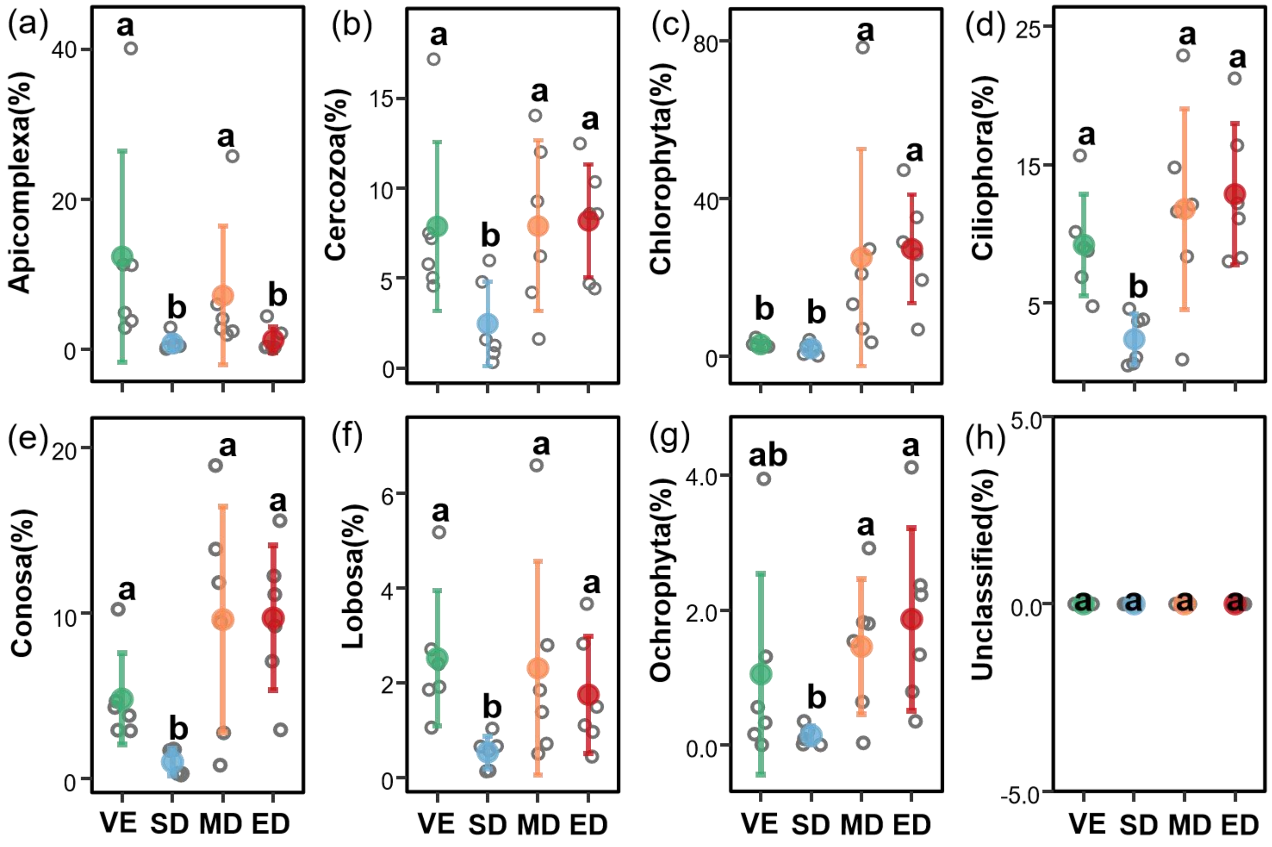

Supplementary Figure 3. Change in the relative abundance of protistan for each phylum.

VE, SD, MD, and ED represent natural vegetation restoration, slight disturbance, moderate disturbance, and extreme disturbance, respectively. Different lowercase letters indicate significant differences among treatments ($p < 0.05$).
